# Artificial intelligence-enhanced interpretation of kidney transplant biopsy: focus on rejection

**DOI:** 10.1097/MOT.0000000000001213

**Published:** 2025-04-01

**Authors:** Alton B. Farris, Jeroen van der Laak, Dominique van Midden

**Affiliations:** a Department of Pathology and Laboratory Medicine; Emory University; Atlanta, Georgia, USA; b Department of Pathology, Radboud University Medical Center, Nijmegen, The Netherlands; c Center for Medical Image Science and Visualization, Linköping University, Linköping, Sweden

**Keywords:** artificial intelligence, digital pathology, kidney biopsy, rejection, transplantation

## Abstract

**Purpose of review:**

The objective of this review is to provide an update on the application of artificial intelligence (AI) for the histological interpretation of kidney transplant biopsies.

**Recent findings:**

AI, particularly convolutional neural networks (CNNs), has demonstrated great potential in accurately identifying kidney structures, detecting abnormalities, and diagnosing rejection with improved objectivity and reproducibility. Key advancements include the segmentation of kidney compartments for accurate assessment and the detection of inflammatory cells to aid in rejection classification. Development of decision support tools like the Banff Automation System and iBox for predicting long-term allograft failure have also been made possible through AI techniques. Challenges in AI implementation include the need for rigorous evaluation and validation studies, computational resource requirements and energy consumption concerns, and regulatory hurdles. Data protection regulations and Food and Drug Administration (FDA) approval represent such entry barriers. Future directions involve the integration of AI of histopathology with other modalities, such as clinical laboratory and molecular data. Development of more efficient CNN architectures could be possible through the exploration of self-supervised and graph neural network approaches.

**Summary:**

The field is progressing towards an automated Banff Classification system, with potential for significant improvements in diagnostic processes and patient care.

## INTRODUCTION

Artificial intelligence (AI) has started to gain widespread use in medicine, including the field of kidney transplantation. Traditionally, assessment of kidney transplant specimens has predominantly depended on the histologic examination of allograft biopsies [1,2]. For some time, data has suggested that the assessment of kidney transplant biopsies requires improvement, particularly with regard to interobserver variability associated with Banff rejection and other scores. This was illustrated years ago in an international study that showed that assessment of Banff lesions did not improve, even after observers were provided with feedback [3]. A subsequent Banff working group focusing predominantly on the assessment of interstitial fibrosis also revealed a lack of agreement among pathologists [4]. Recent advancements in digital and computational pathology have shown novel approaches for time-efficient, quantitative, and reproducible assessment of kidney transplant biopsies that have the potential to support renal pathologists. AI-based computer algorithms can automate objective assessment of cell- and tissue features, and help us to interpret information, generate insights, and provide predictions [5–10]. A convolutional neural network (CNN) is one of the most commonly employed type of deep learning algorithms in kidney pathology; and at an increasing pace, novel networks are being developed [11–13].

CNNs are especially good at segmentation, which is the automated recognition and delineation of various tissue structures like glomeruli, tubules, vessels, and so on. This allows them to serve as a suitable tool to improve the interobserver variability. The Banff Digital Pathology Working Group (DPWG) has put forth efforts to establish image banks, algorithms, and challenges to further promulgate AI and other algorithms [14]. Computer-aided diagnostics will most likely result in more efficient evaluation and standardization. Quantifiable individualized predictions can enhance personalized patient care.

In this review, we provide an overview of AI-enhanced kidney transplant biopsy interpretation, including kidney tissue segmentation and cell detection, rejection diagnosis, and image-based prognosis prediction. We will also discuss challenges and prospects. 

**Box 1:**
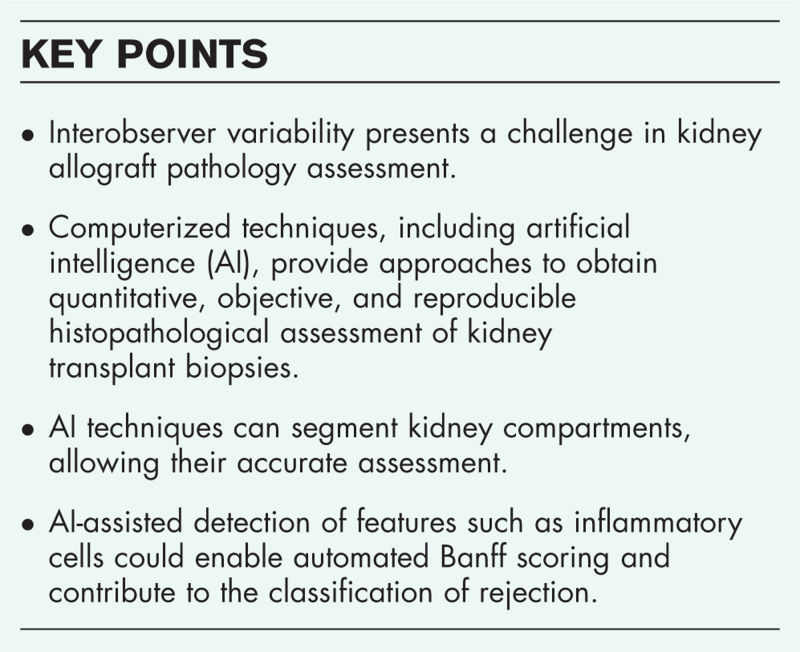
no caption available

## KIDNEY STRUCTURE IDENTIFICATION AND INJURY DETECTION

**FIGURE 1:**
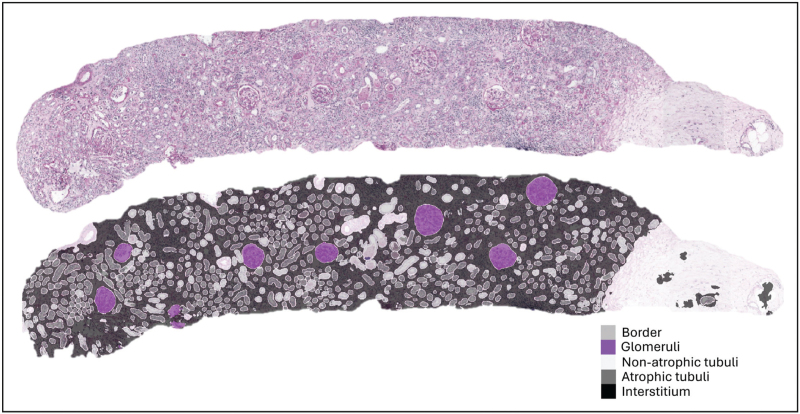
Segmentation of a PAS-stained kidney transplant biopsy on whole-slide level highlighting chronic damage. Nonsclerotic glomeruli are depicted in a different color than sclerotic glomeruli. With the atrophic tubules in dark-grey and interstitium accentuated in black, pathologists can more easily estimate the amount of interstitial fibrosis/tubular atrophy (IFTA).

AI methods can accurately identify the different compartments of the kidney, hence offering insights into the pathologic alterations occurring inside these compartments, such as tubulointerstitial scarring, also known as interstitial fibrosis and tubular atrophy (IFTA) (Fig. 1) [15,16].

**FIGURE 2:**
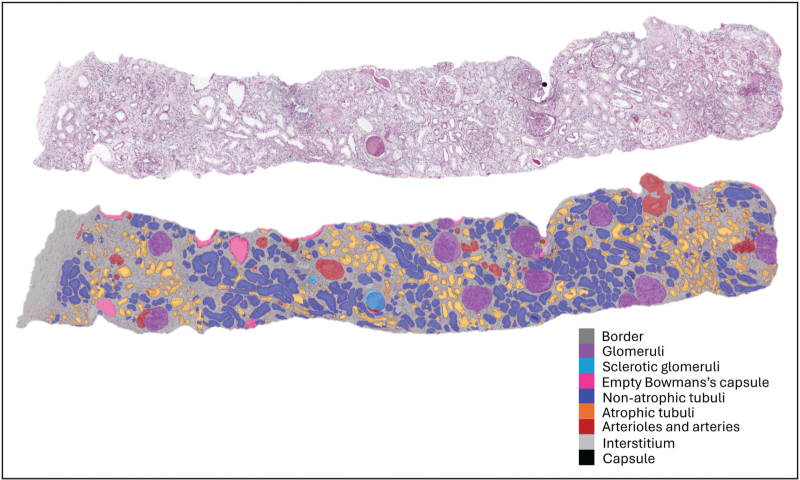
Multiclass segmentation of a PAS-stained kidney transplant biopsy on whole-slide level.

Such methods can serve as a tool to automatically score different Banff Lesion Scores [17]. For example, the number of healthy and sclerotic glomeruli have been identified using CNNs [18–22,23^▪^,^24^,25^▪^]. In 2019, the first kidney histology multiclass segmentation model (Fig. 2) was published [26^▪▪^]. This algorithm was trained on Periodic acid-Schiff (PAS)-stained kidney transplant biopsies and was able to differentiate glomeruli, different types of tubules, interstitium, arteries, and capsule. It showed strong similarity between the model's output and expert-generated ground truth (weighted mean Dice coefficient 0.80–0.84) and correlated well with visual scoring of kidney transplant pathologists.

**FIGURE 3:**
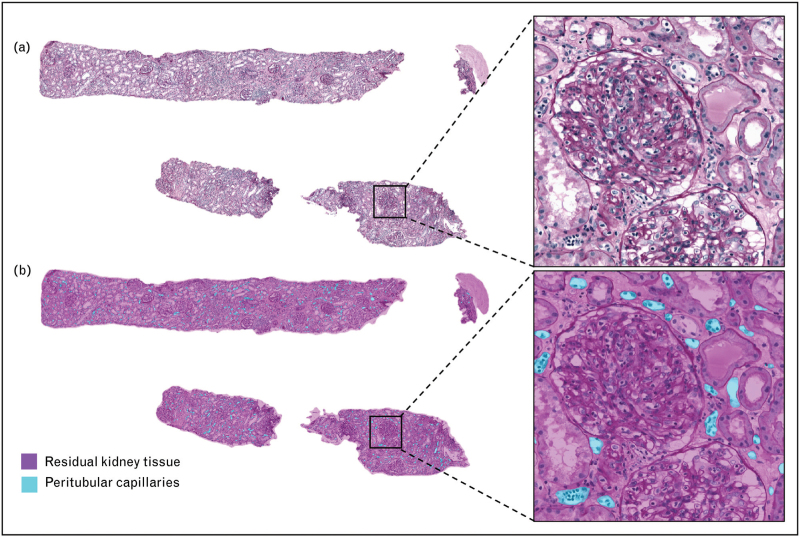
Segmentation of peritubular capillaries in a PAS-stained kidney transplant biopsy. This allows pathologists to quickly assess the degree of peritubular capillaritis.

With the aid of CD3 immunohistochemistry for the detection of T lymphocytes and trichrome staining, CNNs are also able to score the amount of inflammation, interstitial fibrosis, and tubular atrophy [16,27^▪▪^,^28^,29^▪^]. The microvasculature, mostly composed of peritubular capillaries (PTCs), can also be identified using computational methods. This is particularly noteworthy concerning the assessment of peritubular capillaritis in antibody-mediated rejection (Fig. 3) [30^▪^,^31^–^33^].

**FIGURE 4:**
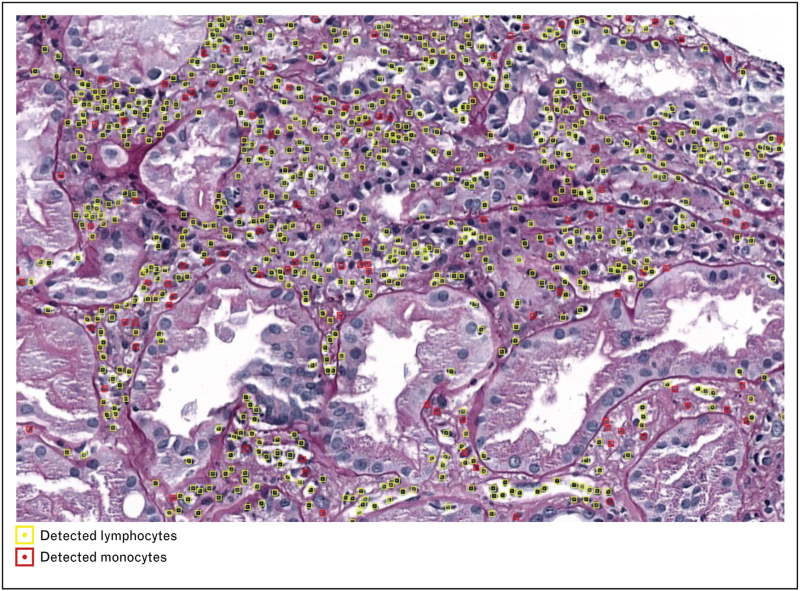
Detection of lymphocytes and monocytes in a PAS-stained kidney transplant biopsy. This is a preliminary result of the MONKEY challenge, which is aimed to develop AI for the detection of inflammatory cells in kidney transplant biopsies. Such a model can be a helpful tool for pathologists to estimate the presence and extent of inflammatory cells in the different kidney compartments in a more time-efficient and reproducible manner. AI, artificial intelligence.

The computational pathology research group (CPG) of the Department of Pathology at Radboud University Medical Center has established the DIAGGRAFT project to benchmark data for accurate, quantitative histological diagnostic assessment of kidney allograft biopsies [14]. Within this project the MONKEY (Machine-learning for Optimal detection of iNflammatory cells in the KidnEY) challenge (https://monkey.grand-challenge.org/) was initiated where participants strive to develop the best-performing model for automated detection of inflammatory cells (Fig. 4). This community-based approach is likely to result in more powerful solutions than single-center developments. By integrating this model with a segmentation model, the group aims to develop an AI tool that automates the Banff Classification.

## ADDITIONAL ARTIFICIAL INTELLIGENCE-POWERED ALGORITHMS

In addition to the more traditional supervised CNN models, new approaches are emerging; and the scope of AI increases. For example, foundational AI models have the ability to combine imaging and nonimaging data such as individual patient characteristics, lab results, molecular data, and medication regimens. Also, semi-supervised and unsupervised approaches are available. Kers *et al.* have applied a semi-supervised/weakly-supervised approach and developed a model to preclassify kidney allograft biopsies into three main broad categories (i.e., normal, rejection, and other diseases) based on histology [34]. This might serve as a potential biopsy triage system. Yoo, Loupy *et al.*[35,36] developed a decision-support system, based on an algorithm covering all Banff classification rules and diagnostic scenarios. This automatically generates diagnoses according to the Banff Classification System. Alexander *et al.* implemented a machine learning classifier to determine mass cytometry spatial biomarkers that can distinguish a variety of kidney allograft inflammatory phenotypes [37^▪▪^].

## DECISION SUPPORT

Decision support tools include the Banff Automation System [35] and the UK Deceased Donor Kidney Transplant Outcome Prediction (UK-DTOP) tool, which utilize AI to provide data to clinicians [38^▪^]. Banff allograft pathology data have been combined with a variety of clinical data in the iBox prediction model, an AI tool for the stratification of kidney allograft performance; and studies have shown that iBox is superior to clinicians in predicting the risk of long-term allograft failure [39]. Machine learning has also been utilized to generate noninvasive virtual donor biopsy findings based on basic donor parameters, providing organ quality assessment for physicians evaluating donor candidacy [40,41].

## CHALLENGES FOR ARTIFICIAL INTELLIGENCE

Despite AI's promising results and advancements in healthcare, its integration into routine practice poses several obstacles. To justify the integration of AI models, rigorous evaluation with robust studies are required to validate the models’ predictive value in which AI-driven quantification (e.g., counting glomeruli, evaluating glomerulosclerosis, IFTA, etc.) is correlated with long-term graft outcomes. Only then can we determine whether AI tools significantly enhance the diagnostic process. This partly has to do with the “black box” issue as DL models often lack transparency in their decision-making process.

CNNs are pivotal in image classification tasks due to their robust feature extraction capabilities. However, executing such computationally demanding computer vision models necessitates certain system requirements and may have a negative effect on our planet in terms of unnecessary energy consumption [42,43]. This calls for innovative solutions such as efficient AI model architectures. Regional and global regulatory aspects like Food and Drug Administration (FDA) approval poses another obstacle for implementation [44,45]. For example, the EU's General Data Protection Regulation hinders easy implementation as it stipulates that “the data subject shall have the right not to be subject to a decision based solely on automated processing”.

Ethical challenges exist in the application of AI, particularly concerning algorithmic bias and equity. Models trained on nonrepresentative datasets could perpetuate racial disparities, embed historical inequities into clinical workflows, and misclassify marginalized populations. A biased algorithm developed for transplantation could lead to delayed transplantation for some populations compared to others. This risk could be mitigated through increased awareness of training cohort demographics and interdisciplinary collaboration [8,46,47^▪^,^48^–^52^].

## THE ROAD AHEAD

With the introduction of artificial intelligence in pathology for image analysis and the rise of CNNs, the field is evolving and becoming more heterogeneous. Researchers and enterprises are seeing more cost-effective, efficient, and transparent solutions emerge. By developing lightweight and more efficient CNN architectures, the computational requirements reduce without compromising accuracy. EfficientNet and Inception serve as exemplars [53]. Self-supervised [54] and graph neural network [55] methodologies are also being explored. The next wave of innovation involves the integration of CNNs with other AI modalities, especially generative models and multimodal systems. These hybrid AI models can process both visual and textual data, creating new opportunities for applications like multimodal research. TITAN is a multimodal whole-slide foundation model developed by Faisal Mahmood's team and trained on >330 000 pathology slides. They used a diverse set of neoplastic, infectious, and inflammatory cases and corresponding captions synthetically generated via PathChat [56]. The recently introduced DeepSeek chatbot possibly illustrates how more efficient methods could revolutionize the AI industry [57,58].

Recently there is increased interest in xenotransplantation as a possible method to solve the organ shortage problem [59–61]. As xenotransplantation is slowly becoming a realistic strategy, AI tools might help efficiently disseminate expert knowledge on xenotransplantation pathology so that enhanced interpretation is available for clinical trials. Similarly, AI will likely be quite crucial in the application of new immunosuppression strategies and regenerative medicine [62].

## CONCLUSION

AI-based tools hold great promise with regard to kidney transplant pathology interpretation. Studies have demonstrated their accuracy in segmenting kidney structures, detecting abnormalities, and diagnosing rejection in a more objective and reproducible manner. Ancillary techniques can be combined with AI tools to provide enhanced data and diagnoses for precision patient care. By integrating segmentation models with detection models (e.g., inflammatory cells) the field is going toward an automated Banff Classification System. Expanding this further with additional AI modalities like foundational models has the potential to significantly improve the diagnostic process.

However, unresolved issues persist in this field. For example, the computational resources necessary for running these algorithms demand energy, so the sustainability of utilizing these resources will need to be considered in the future [63]. In addition, robust studies that rigorously evaluate AI models with long-term graft outcomes are needed to justify the integration of AI in routine practice. Also, regulatory aspects pose a challenge; and methods to combat bias should be considered. Still, AI-based tools hold great promise. The advancement of novel AI-assisted techniques and modalities through multidisciplinary collaboration of computer engineers, kidney specialists, and nephropathologists will address existing challenges and ensure a promising future for AI applications in nephropathology.

## Acknowledgements

*We would like to thank the Dutch Kidney Foundation for funding Dr van Midden in the Kolff+ program (grant number 21OK+012).*

### Financial support and sponsorship

*None.*

### Conflicts of interest

*Alton Brad Farris's institution, Emory University, receives National Institute of Health (NIH) funding for his involvement in a collaboration with Kitware, Inc.*

*Jeroen van der Laak was a member of the advisory boards of Philips, the Netherlands and ContextVision, Sweden, and received research funding from Philips, the Netherlands, ContextVision, Sweden, and Sectra, Sweden in the last five years. He is chief scientific officer (CSO) and shareholder of Aiosyn BV, the Netherlands.*

## REFERENCES AND RECOMMENDED READING

Papers of particular interest, published within the annual period of review, have been highlighted as:

▪ of special interest
▪▪ of outstanding interest
